# iTRAQ-Based Proteomics Reveals Novel Members Involved in Pathogen Challenge in Sea Cucumber *Apostichopus japonicus*

**DOI:** 10.1371/journal.pone.0100492

**Published:** 2014-06-20

**Authors:** Pengjuan Zhang, Chenghua Li, Peng Zhang, Chunhua Jin, Daodong Pan, Yongbo Bao

**Affiliations:** 1 Department of aquaculture, Ningbo University, Ningbo, Zhejiang Province, P.R China; 2 Department of Aquatic Germplasm Resources, Zhejiang Wanli University, Ningbo, Zhejiang Province, P.R China; Uppsala University, Sweden

## Abstract

Skin ulceration syndrome (SUS) is considered to be a major constraint for the stable development of *Apostichopus japonicus* culture industries. In this study, we investigated protein changes in the coelomocytes of *A. japonicus* challenged by *Vibrio splendidus* using isobaric tags for relative and absolute quantification (iTRAQ) over a 96 h time course. Consequently, 228 differentially expressed proteins were identified in two iTRAQs. A comparison of the protein expression profiles among different time points detected 125 proteins primarily involved in response to endogenous stimuli at 24 h. At 48 h, the number of differentially expressed proteins decreased to 67, with their primary function being oxidation reduction. At the end of pathogen infection, proteins responsive to amino acid stimuli and some metabolic processes were classified as the predominant group. Fifteen proteins were differentially expressed at all time points, among which eight proteins related to pathologies in higher animals were shown to be down-regulated after *V. splendidus* infection: paxillin, fascin-2, aggrecan, ololfactomedin-1, nesprin-3, a disintegrin-like and metallopeptidase with thrombospondin type 1 motif (Adamts7), C-type lectin domain family 4 (Clec4g) and n-myc downstream regulated gene 1 (Ndrg1). To gain more insight into two SUS-related miRNA (miR-31 and miR-2008) targets at the protein level, all 129 down-regulated proteins were further analyzed in combination with RNA-seq. Twelve and eight proteins were identified as putative targets for miR-31 and miR-2008, respectively, in which six proteins (5 for miR-31 and 1 for miR-2008) displayed higher possibilities to be regulated at the level of translation. Overall, the present work enhances our understanding of the process of *V. splendidus*-challenged sea cucumber and provides a new method for screening miRNAs targets at the translation level.

## Introduction

Sea cucumber *Apostichopus japonicus*, famous for its superior nutritive value and supposed medicinal properties, has become one of the most important aquaculture species in China, and its aquaculture has grown rapidly since the 1980s. However, the intensification and rapid expansion of *A. japonicus* farming has also led to the occurrence of various diseases [1].Skin ulceration syndrome (SUS) is one of the most common diseases with a high mortality of 90%-100% and has become a limiting factor in the sustainable development of this industry [2]. Some reports have demonstrated that the pathogens responsible for the outbreak of skin ulceration include aspherical virus [3], *Vibrio splendidus* [4] and *Pseudomonas spp* [5]. Among these pathogens, *V. splendidus* has been widely accepted as one of the major pathogens. To date, many efforts have been made to study the pathogenic progress of SUS outbreak caused by *V. splendidus*, but the intrinsic mechanism still requires further investigation.

MicroRNAs (miRNAs) are a type of approximately 22 nt-sized small noncoding RNAs that exist in diverse organisms [6]. It has been confirmed that in plants, miRNA-target interactions are often within the coding region and nearly perfectly complementary, which triggers mRNA cleavage. By contrast, animal miRNA/target duplexes generally are interrupted by gaps and mismatches and occur in the 3′UTR of mRNAs. Pioneering genetic studies in *Caenorhabditis elegans* have identified that lin-4 can inhibit the translation of lin-14 and lin-28 mRNA without affecting the cellular level of lin-14 and lin-28 [7] [8]. Recently, miRNAs have emerged as key regulators of a broad spectrum of cellular activities, including immune response [9], insulin secretion [10], and viral replication [11]. In miRNA research, the identification of the targets of individual miRNAs is of utmost importance. Our understanding of the molecular mechanisms by which individual miRNAs modulate cellular functions remains incomplete until a full set of miRNA targets is identified and validated. Since miRNAs can function through partial or full complementary base pairing to their target messenger RNAs (mRNAs), resulting in translational inhibition or mRNA degradation [12], it seems to be feasible to identify the putative targets of miRNAs by detecting the cellular level of mRNAs or proteins from the same biological sample. In our previous work, miR-31 and miR-2008 were demonstrated to be involved in SUS outbreak [13], and their candidate targets were also predicted by RNA-seq analysis by miRanda toolbox based on the reverse expression pattern between miRNA and mRNA [14]. However, negative expression correlations were not detected by qRT-PCR for most of their putative targets at the mRNA level in bacteria challenged samples, indicating that the targets might be regulated at the protein level without affecting mRNA abundance. Therefore, proteomics is a suitable method to reveal the full spectrum of miRNA targets and quantify the contribution of translational repression by miRNAs because it provides a rapid and comprehensive evaluation of protein profiles in complex protein samples. Over the past few decades, many proteomic platforms have been developed for the qualitative and quantitative characterization of protein mixtures and post-translational modifications, such as 2D gel-MS [15], LC MS/MS [16]. Currently, isobaric tags for relative and absolute quantification (iTRAQ) has unique advantages over other conventional proteomics techniques because iTRAQ identifies and quantifies many proteins from specific biological environments using labeled peptides identifiable by sensitive mass spectrometers [17]. iTRAQ analysis is further strengthened by using robust bioinformatic tools and statistical analyses to support observations [18]. Using the infection model and iTRAQ approach, many researchers have made great advances in identifying proteins involved in the pathogenic process [19] [20]. Moreover, the iTRAQ approach has also been successfully employed for the identification of miRNA targets in other species [21] [22]. However, the identification of proteins by tandem mass spectrometry requires reference protein databases that are only available for model species. Because of the recent contributions to the transcriptomic characterization of the *A. japonicus* immune system, including transcriptome analysis of diseased [14] and LPS-challenged *A. japonicus* [23], a protein database for mass spectrometry-based identification of non-model organisms has been generated. Meanwhile, NMR-based metabonomics have been performed to explore the metabolic changes in the muscle tissues of pathogen-challenged and diseased *A. japonicus*, providing a comparative understanding of the metabolic profiles under different conditions [24]. To understand the intrinsic pathogenic mechanism, we analyzed protein expression patterns over a 96 h time course pathogen infection to identify proteins and peptides changes in response to *V. splendidus* challenge and reveal miRNA targets at the translational level, thus increasing our knowledge of cellular pathways important for infection and pathogenesis.

## Materials and Methods

### Ethics statement

The sea cucumbers (*A. japonicus*) here are commercially cultured animals, and all the experiments were conducted in accordance with the recommendations in the Guide for the Care and Use of Laboratory Animals of the National Institutes of Health. The study protocol was approved by the Experimental Animal Ethics Committee of Ningbo University, China.

### Experimental animals and conditions

One hundred healthy adult sea cucumbers *A. Japonicus* (165±23 g) were obtained from Bowang Aquaculture Company (Ningbo, China) and evenly assigned to four tanks randomly. The animals were then acclimatized in aerated natural seawater (salinity 25 psu, temperature 16°C) for three days prior to be treated. *V. splendidus* were initially isolated from a skin ulceration diseased sea cucumbers and identified by 16S rRNA. The confirmed bacteria were cultured in liquid 2216E broth (Tryptone 5 g L-1, yeast extract 1 g L-1, pH 7.6) at 28°C, 140 rpm and centrifuged at 1,000 g for 5 min to harvest the bacteria. Live *V. splendidus* were then re-suspended in filtered seawater (FSW). For the challenge experiments, one tank used as a control, and the other three tanks were immersed with high density of *V. splendidus* with a final concentration of 10^7^ CFU mL^−1^. After being challenged for 24 h, the coelomocytes were collected from control and challenged group to confirm the exist of V. splendidus by 16S rRNA PCR. The sea cucumbers were then dissected and coelomic fluids were collected from five individuals in each tank, and approximately 50 ml of coelomic fluids were gathered at 0, 24, 48 and 96 h, respectively. Of which, 45 mL was severed as sample for iTRAQ analysis, and 5 mL for RNA quantify. The coelomic fluids was then centrifuged at 1,000 g for 5 min to harvest the coelomocytes and the coelomocytes were then stored at −80°C before protein and RNA extraction extraction.

### Protein extraction, quantization, digestion and iTRAQ labeling

Two biological replicates of each group were prepared for the proteomics experiments. Briefly, the total protein of each sample was grinded to powder with liquid nitrogen and dissolved in lysis solution [9 M Urea, 4% CHAPS, 1%DTT, 1%IPG buffer (GE Healthcare)]. The mix was incubated at 30°C for 1 hour and centrifuged at 15,000 g for 15 min at room temperature. The supernatant was collected and quantified by the Bradford method [25].

For each sample, 100 µg of protein was dissolved in a dissolution buffer (AB Sciex, Foster City, CA, USA). After being reduced, alkylated and trypsin-digested, the samples were labeled following the manufacturer's instructions for the iTRAQ Reagents 8-plex kit (AB Sciex). Samples taken at 0, 24, 48 and 96 h were each labeled with iTRAQ reagents with molecular masses of 113, 114, 115, and 116 Da, respectively. Additional independent biological replicates were labeled with other reagents with molecular masses of 117, 118, 119, and 121 Da. After labeling, all samples were pooled and purified using a strong cation exchange chromatography (SCX) column by Agilent 1200 HPLC (Agilent) and separated by liquid chromatography (LC) using a Eksigent nanoLC-Ultra 2D system (AB SCIEX). The LC fractions were analyzed using a Triple TOF 5600 mass spectrometer (AB SCIEX). Mass spectrometer data acquisition was performed with a Triple TOF 5600 System (AB SCIEX, USA) fitted with a Nanospray III source (AB SCIEX, USA) and a pulled quartz tip as the emitter (New Objectives, USA). Data were acquired using an ion spray voltage of 2.5 kV, curtain gas of 30 PSI, nebulizer gas of 5 PSI, and an interface heater temperature of 150°C. For information dependent acquisition (IDA), survey scans were acquired in 250 ms and as many as 35 product ion scans were collected if they exceeded a threshold of 150 counts per second (counts/s) with a 2^+^ to 5^+^ charge-state. The total cycle time was fixed to 2.5 s. A rolling collision energy setting was applied to all precursor ions for collision-induced dissociation (CID). Dynamic exclusion was set for ½ of peak width (18 s), and the precursor was then refreshed off the exclusion list.

### Protein Identification and Quantification

The iTRAQ data were processed with Protein Pilot Software v4.0 against the *A. japonicus* database using the Paragon algorithm [26]. Protein identification was performed with the search option of emphasis on biological modifications. The database search parameters were the following: the instrument was TripleTOF 5600, iTRAQ quantification, cysteine modified with iodoacetamide; biological modifications were selected as ID focus, trypsin digestion. For false discovery rate (FDR) calculation, an automatic decoy database search strategy was employed to estimate FDR using the PSPEP (Proteomics System Performance Evaluation Pipeline Software, integrated in the ProteinPilot Software). The FDR was calculated as the number of false positive matches divided by the number of total matches. Then, the iTRAQ was chosen for protein quantification with unique peptides during the search, and peptides with global FDR values from fit less than 1% were considered for further analysis. Within each iTRAQ run, differentially expressed proteins were determined based on the ratios of differently labeled proteins and p-values provided by Protein Pilot; the p-values were generated by Protein Pilot using the peptides used to quantitate the respective protein. Finally, for differential expression analysis, fold change was calculated as the average ratio of 114/113 and 118/117 at 24 h, 115/113 and119/117 at 48 h, 116/113 and 121/117 at 96 h, respectively. and proteins with a fold change of >1.5 or <0.67 and p value less than 0.05 were considered to be significantly differentially expressed.

### GO and KEGG pathway enrichment analysis

For GO and KEGG pathway enrichment analysis, the homology search was first performed for all query protein matches with blastp against the *Mus musculus* protein database. The E-value was set to < 1e-10, and the top 10 best hits for each query sequence were taken. Among the 10 best hits, the hit with the best identity to the query was picked as homologous. The GO analysis was performed with different mapping steps to link all blast hits to the functional information stored in the Gene Ontology database using the DAVID toolkit. Public resources such as NCBI, PIR and GO are used to create links with protein IDs and corresponding gene ontology information. All annotations are associated to an evidence code that provides information regarding the quality of this functional assignment. For KEGG Pathway enrichment analysis, the pathway enrichment was performed using annotated proteins in the query dataset against the KEGG database, and Cytoscape was used for protein-protein interaction network construction.

### miRNA target prediction

miRNA target prediction was performed only for down-regulated proteins. The corresponding mRNA sequences were acquired from RNA-seq analysis [14]. Computational identification of miR-31 and miR-2008 targets was performed using the miRanda toolbox to search complementary regions between miRNA and the 3′UTR of mRNA with default parameters of S>90 (single-residue pair scores) and ΔG<-17 kal/mol.

### miRNA and RNA quantification analysis

Total RNA was extracted with the RNAiso plus reagent (Takara, Japan) following the manufacturer's instructions. The SYBR green qRT-PCR assay was used for miRNA and mRNA quantification in identical samples. In brief, 500 ng RNA containing miRNAs was polyadenylated by poly(A) polymerase and converted to cDNA by reverse transcriptase using the miScript Reverse Transcription Kit (Qiagen, Germany). For miRNA analysis, the qRT-PCR was performed using the miScript SYBR Green PCR kit (Qiagen, Germany) with the manufacturer-provided miScript Universal primer and the miRNA-specific forward primers (for mRNA, the primers were gene-specific forward and reverse primers) in a Rotor-Gene Q 6000 Real-time PCR detection system (Qiagen, Germany).

The miRNA-specific primers were designed based on the miRNA sequences obtained from [13] and the gene-specific primers were designed based on the mRNA sequences obtained from [14]. All the primers used in qRT-PCR were shown in table 1. Each reaction was performed in a final volume of 20 µl containing 2 µl of the cDNA, 10 µM of each primer, 6 µl RNase-free water and 10 µl SYBR Green PCR Master mix (Qiagen). The amplification profile was: denaturation at 94°C for 15 min, followed by 40 cycles of 94°C for 15 s, 60°C for 30 s and 70°C for 30 s, in which fluorescence was acquired. At the end of the PCR cycles, melting curve analyses were performed. Each sample was run in triplicates for analysis. The expression levels of miRNAs were normalized to RNU6B[27], and the expression levels of mRNAs were normalized to 18S rRNA[28].

**Table 1:** Primers used in qRT-PCR.

Gene name	Forward primer (5′-3′)	Reverse primer (5′-3′)
Spu-miR-31	AGGCAAGATGTTGGCATAGCT	Qiagen miScript universal primer
Spu-miR-2008	ATCAGCCTCGCTGTCAATACG	
Actr3	CTACCATGTTCAGGGATTTCGG	GGTGAGAGATGACCTCTGTTTCG
Clec4g	CCAACGGGAACCAAACAAT	TCGCAAACGCCAAACCTAAC
Cndp2	CTGTCGGATGGTTGGGATACTG	CATTCAGCCAGGCAAGAACG
Coro6	GCCATACGCATTCGTCATTTG	GATTGTTTCTCCTTCCTCCTCC
Gnb2	GCAAACATTTACCGGACACGAG	CAAGTAGCAAACGGCCACTCC
H1f0	CCATCGCCGACCTGAATG	AGCCTGAAGGTGCCACTCG
Hnrnpa1	GAGGTGATTATGCCAATGTCTGC	CACCACCGCCTCTTCCTTCT
Hnrnpl	CACGCAAGTCATCCACAAAGTG	AAACATCCCTCCCATTCAGCC
HSP90b1	TACTCCGTTCTGGGTTCATGC	TCTTCCTCTTCTGGTTCCTCCTC
Hspa8	GTGCCAACCCATCATCACCA	ACCTGCACCTGGAAACCCTC
Kiaa0196	GTTGATTGGCGATACCAAGCAC	GACCTGTTCCCGCAGTTGAC
Myo10	GCCGTGGGTGCTAAGATGGT	GAGTTCTGCCTCCTGTGGACAAT
Naca	AACAGTTCCGACCACAGGAAC	CTCTTCCAACCCACCATCATC
Nnt	AGGTAAGCCTATGGCGATTGAG	CATCTCTGCGATTGGATACTGG
Plekhd1	AGGCCGAGGATTCCTTGTG	CTCTGCGGGAGATTGTGATGA
Rpl12	CTGTTGGTGGAGAAGTTGGAGC	CTGCCTGTTCTGGATGGTCAAC
Slc25a5	GCAGCTTACTTCGGGTTCTATG	GATCTTCCTCCAGCAGTCCAG
Tgds	CTCAAGGCTCAATCACCAAGC	CGACCAAGGGAGGATGTGTT
Yes1	CGAAGATTCCGAATACACCGC	AGGACACCATAGGACCACACGT

The 2^−ΔΔCt^ method was used to analyze the expression levels of both miRNA and mRNA, and the obtained values represented the n-fold difference relative to the control (untreated samples). The data are presented as relative expression levels (means±S.D, n = 3), and all experimental data were subjected to one-way Analysis of Variance (ANOVA) followed by multiple Duncan tests to determine differences in the mean values among the controls. Significant differences between the treated and corresponding control groups at each time point are indicated with one asterisk for P<0.05 and two asterisks for P<0.01. The error bars in the graphs represent standard deviations.

## Results

### Overview of changes in host proteome induced by *V. splendidus* infection

**Figure 1:**
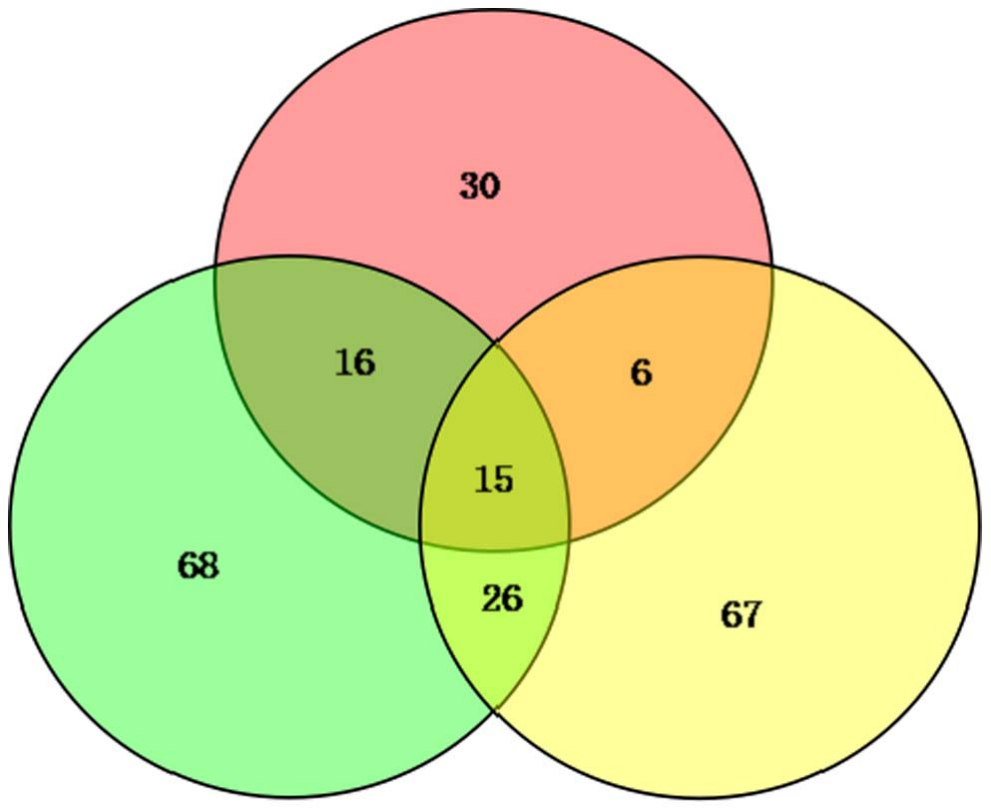
Changed proteome distribution between different time points, Venn diagram showing unique and shared proteins between time points.

Compared with the control group, the *V. splendidus* were detected in the challenged groups. And the samples were then used for iTRAQ analysis. In conclusion, we describe global proteome changes in the *A. japonicus* coelomocytes during the short-term course of *V. splendidus* infection. We identified 2793 distinct proteins, of which 2049 were identified and quantified reliably at a global false discovery rate (FDR) of 1%. Compared with the control group, 228 identified proteins had significant changes in expression at different time points, among which 15 were observed to be differentially expressed at all examined time points (Fig. 1). A total of 99 proteins displayed increased expression trends (fold change >1.5, p≤0.05), and 129 proteins displayed decreased expression levels (fold change <0.67, p≤0.05) compared with the control group (Table S1). Notably, eight proteins related to pathology in higher animals exhibited down-regulated expression after *V. splendidus* infection: paxillin, fascin-2, nesprin-3, aggrecan, ololfactomedin-1, a disintegrin-like and metallopeptidase with thrombospondin type 1 motif (Adamts7), c-type lectin domain family 4 (Clec4g) and N-myc downstream regulated gene 1 (Ndrg1).

### Proteomic analysis at each time point after pathogen infection

**Figure 2:**
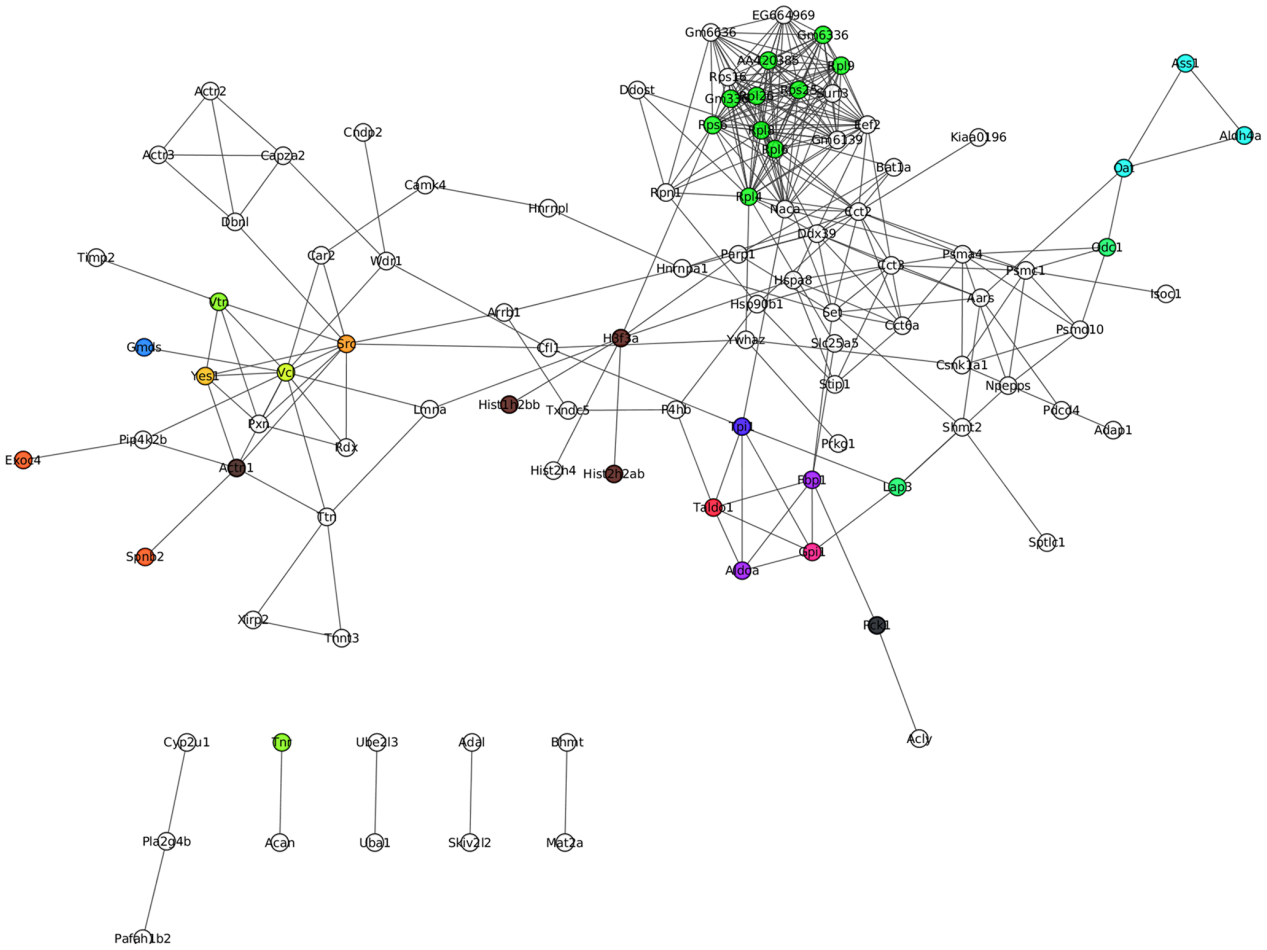
Protein-protein interactions were obtained by the string database. Interaction maps were created by cytoscape. The snapshot shows direct interactions found in these differentially expressed proteins from each time point.

Among these differentially expressed proteins, there were 125, 67 and 114 proteins identified in pathogen-challenged *A. japonicus* at 24, 48 and 96 h, respectively, compared with the control group. Of these, 67, 30, and 67 proteins were uniquely identified at 24, 48, 96 h post infection, respectively (Table S2). Some well-documented immune-related proteins were also included into this group. Glutathione S-transferase were uniquely expressed at 24 h and sharply increased to 3.587-fold compared to control group. However, ficolin B and guanine nucleotide binding protein were down-regulated their expression to 0.026-fold and 0.171-fold at 48 h and 96 h, respectively. More importantly, some apoptosis-related proteins, such as sarcoma oncogene (Src), vitronectin and vinculin, displayed time-dependent depressed expression (Table S2). Protein-protein interaction (PPI) analysis further indicated a diversified functional network of these novel proteins at different time points (Fig. 2).

### GO and KEGG analysis of the differential expression proteins

**Figure 3:**
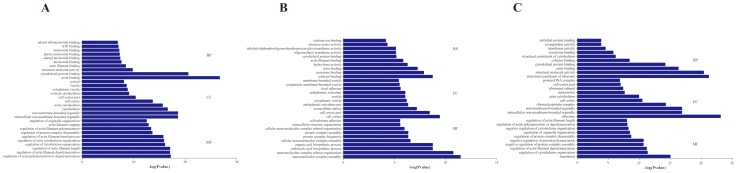
GO enrichment analysis of differentially expressed proteins at each time point. A: 24 h; B: 48 h; C: 96 h.

**Figure 4:**
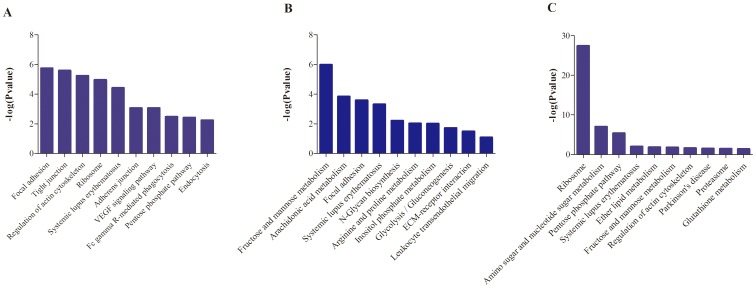
KEGG pathway enrichment analysis of differentially expressed proteins at each time point. A: 24 h; B: 48 h; C: 96 h.

GO enrichment demonstrated that 80, 44, and 26 protein categories were enriched in the Biological process (BP), Cellular component (CC) and Molecular function (MF) categories, respectively (Table S3). Among these, actin filament depolymerization, intracellular non-membrane-bounded organelle, and actin binding were the most abundant categories in BP, CC and MF, respectively. The differentially expressed proteins were predominately binding proteins involving in cytoskeleton organization processes at 24 h post infection. At 48 h, differentially expressed proteins were also primarily binding proteins involving in protein complex assembly for extracellular structure organization, and at 96 h, differentially expressed proteins were predominately proteins related to ribosomal assembly and regulating the actin cytoskeleton (Fig. 3). Further KEGG pathway enrichment revealed that these proteins were mainly involved in Focal adhesion and cytoskeleton regulation pathways (24 h); various sugar metabolisms and related to protein complex assembly (48 h); and ribosome, amino sugar and nucleotide sugar metabolism (96 h) (Fig. 4).

### Target prediction of miR-31 and miR-2008

By integrating these results with our RNA-seq and miRanda toolbox analyses, 12 and 8 proteins were identified as putative targets for miR-31 and miR-2008 from the 129 down-regulated proteins, respectively (Table S4). Heat shock protein 90 (Hsp90b1) was the only common target for both of these miRNAs.

### Quantitative analysis of miRNAs and mRNAs

**Figure 5:**
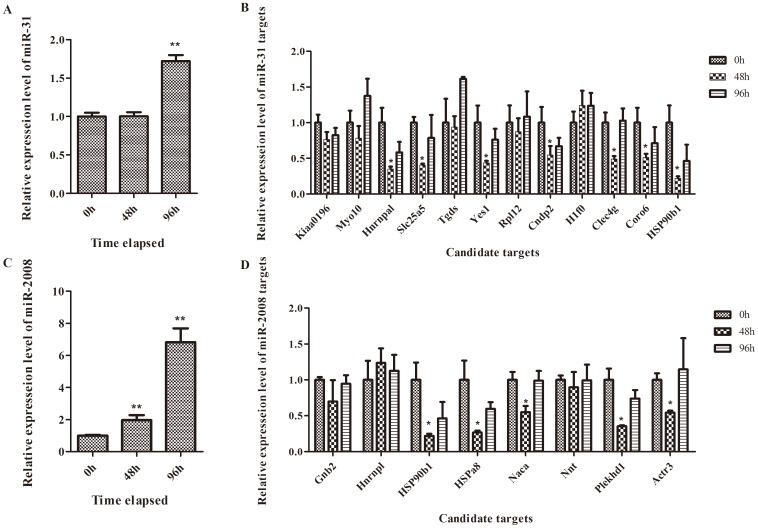
Time-course expression patterns of miRNAs and its putative targets of *A. japonicus* upon *V. splendidus* treatment. Hnrnpa1: Heterogeneous nuclear ribonucleoprotein L; Yes1: Yamaguchi sarcoma viral oncogene homolog 1; Cndp2: CNDP dipeptidase 2;Coro6: Coronin 6; Hspa8: Heat shock 70 kDa protein 8; Plekhd1: PH domain-containing family D member 1; Slc25a5: solute carrier family 25.

The expression patterns of these two miRNAs (miR-31 and miR-2008) and their 20 putative targets were examined by quantitative PCR and the results are shown in Fig. 5. miR-31 and miR-2008 were both significantly up-regulated after pathogen infection. For the targets of miR-31, five genes-WASH complex subunit strumpellin (Kiaa0196), myosin X (Myo10), TDP-glucose 4,6-dehydratase (Tgds), 60S ribosomal protein L12 (Rp112) and H1 histone family, member 0 (H1f0)—displayed unchanged expression at the mRNA level. Guanine nucleotide binding protein beta 2 (Gnb2), nascent polypeptide-associated complex alpha (Naca), nicotinamide nucleotide transhydrogenase (Nnt) and heterogeneous nuclear ribonucleoprotein A1 (Hnrnpl) maintained constant expression of the miR-2008 targets.

## Discussion

### Differentially expressed proteins between pathogen-challenged and control groups

In the current study, we used the iTRAQ method to screen proteins potentially involved in the host-pathogen interaction, and 228 proteins were identified as differentially expressed combined across all time points. However, contrary to our previous knowledge regarding immunogenic factors in invertebrates, most of these proteins have not been previously well studied. Functional annotation of these shared and unique proteins provides new insight into the pathophysiology of *V. splendidus* infection in *A. japonicus*. Of these 15 commom differential proteins in all examined time points, 13 were down-regulated (paxillin, aggrecan, Tgds, Adamts7, fascin-2, olfm-1, Clec4g, nesprin-3, Ndrg1, histone cluster 1 (Hist1h4f), histone cluster 1 (Hist1h2bb), mucin 5ac, H1 histone family) and 2 were up-regulated [titin and c-maf inducing protein (Cmip)]. Most of these down-regulated proteins are signal integration and transduction-related components and some are even involved in the activation of the immune response pathway. Given the commonalities of these shared proteins in pathogen-challenged samples, we propose that the down-regulation of these cell communication-related proteins may contribute to the immune evasion of *V. splendidus*.

### Unique proteins revealed the temporal and spatial host-pathogen interaction progress

The biological roles of unique proteins identified from each comparison (Fig. 1) were further examined to understand the changes occurring at each time point. From the perspective of temporal progress of host-pathogen interaction, there was a function-biased conversion from 24 h to 96 h post-infection. In the early phase after *V. splendidus* infection (24 h), the unique proteins predominantly had actin or cytoskeletal protein binding activities and were involved in actin polymerization or depolymerization and cytoskeleton organization processes. At 48 h, the function of the uniquely differentially expressed proteins included calcium ion, vitamin or metal ion binding activities involved in oxidation-reduction and some metabolic processes. Finally, in the late phase of infection (96 h), the function of its unique proteins were transferase activities, exopeptidase activities and cofactor or coenzyme binding activities involved in translation, protein folding and some catabolic processes. The different distributions of functions among these unique proteins at each time point demonstrated a temporal process initiating from environmental information management to the final stress respondent.

On the other hand, diversified immune- and apoptosis-related proteins were also identified at each time points (Table S2), indicating that modulating immune system and apoptosis pathway were the utmost event in this host-pathogen interaction process. A number of effectors like lectin, lysozyme and Toll had been addressed to be involved into *V. splendidus* challenged sea cucumber. In the present study, diverse expression profiles of immune-related genes were detected between 24 h and other two time points. The up-regulation of Gstt1 at 24 h might result from the host immune response upon pathogen attack. As the GST family had been known as phase II detoxification enzymes which involved in response to pathogen attack, oxidative stress, and heavy-metal toxicity [29]. And the gene expression pattern of theta-class of GST in *Vibrio tapetis* and LPS challenged molluscan *Ruditapes philippinarum* also shown the similar tendency marked as the sharply raised expression level at 6 h and 24 h post infection [30]. The Ficolins were serum complement lectins which were able to recognize pathogen-associated molecular patterns (PAMPs) and trigger the activation of immune system by initiating activation of complement pathway or stimulating secretion of the inflammatory cytokines [31]. Another down-regulated immune related protein, Gnb2 belong to the G protein family, which function as G protein signaling to modulate cell motility and killing of invading pathogen. The significantly down-regulation of these two factors might represent negative modulation of the innate immune response as the insufficiency of Fcnb and Gnb2 might result in higher susceptibility to infection in individuals.

However, apoptosis related molecules like Src, vitronectin and vinculin were scarcely investigated in sea cucmber. Src is the canonical member of the non-receptor family of tyrosine kinases, which could inhibit apoptosis through the Erk1/2- dependent degradation of the death accelerator Bik [32]. Previous study also demonstrated that in the human primary macrophages, the Src tyrosine kinase activity were essential for the inflammasome activation during influenza A virus infection [33]. Vitronectin was considered to modulate neutrophil adhesion, chemotaxis, to contribute to neutrophil-associated proinflammatory processes [34], and to inhibit neutrophil apoptosis through activation of integrin-associated signaling pathways [35]. Vinculin was known to regulate extracellular signal-regulated kinase (ERK) by modulating the accessibility of paxillin for FAK interaction, thus controlling the survival and motility of cells which were critical to metastasis [36]. But recent study also shown that vinculin was emerging as a regulator of apoptosis and Shigella entry into host cells [37] [38]. What is more important is that Pxn served as a “hub” protein for Src, vitronectin and vinculin, indicating a potential apoptosis related signal transportation role of paxillin under bacterial infectious conditions.

### Metabolism and fuel usage during pathogen infection period

Recently, an NMR-based metabonomics study was conducted to explore the metabolic changes in muscle tissues of pathogen-challenged *A. japonicus*. This study observed that the pathogen did not induce obvious biological effects in *A. japonicus* samples 24 h after infection. Enhanced energy storage and immune responses were observed in *V. splendidus*-challenged *A. japonicus* samples at 48 h, marked by increased glucose and branched chain amino acids, respectively. Finally, infection of *V. splendidus* induced significant increases in the energy demand of *A. japonicus* samples at both 72 and 96 h, confirmed by decreased glucose and glycogen and increased ATP levels [24]. Because of technology restrictions, it is difficult to examine metabolic changes in pathogen-challenged coelomocytes. However, by reference to the metabonomics in the muscle tissues of *V. splendidus*-challenged samples and combined with our proteome analysis, we can infer metabolic changes in coelomocytes.

At 24 h post-infection, down-regulation of glucose phosphate isomerase-1 and transaldolase-1 in the pentose phosphate pathway will result in the accumulation of α-D-Glucose-6P and β-D-Fructose-6P and deficiency of D-Glyceraldehyde-3P, which may increase the concentration of pyruvate and inhibit the glycolysis pathway. Excessive pyruvate will promote the biosynthesis of leucine, isoleucine and valine, resulting in increased levels of glucose and branched amino acids. At 48 h post-infection, the up-regulation of triosephosphate isomerase-1 in the fructose and mannose metabolism pathway will increase the level of intracellular Glyceraldehyde-3P, which will accelerate the glycolysis pathway. Moreover, the up-regulation of ornithine aminotransferase and argininosuccinate synthetase-1 in arginine and proline metabolism will facilitate the urea cycle, which will further promote alanine metabolism. In addition, the typical decrease of protein synthesis-related proteins, such as Ribosomal proteins L4 and L8, that was observed at 96 h post infection may have resulted from decreased cellular amino acid levels and increased energy demand.

### Coupling transcriptional and post-transcriptional miRNA regulation in host immune response

In our previous study, miR-31 and miR-2008 were identified from diseased individuals [13]. It has been widely accepted that animal miRNAs exert most of their silencing through the inhibition of translation, rather than through mRNA degradation of their targets, because of the low overall degree of sequence complementarity that animal miRNAs share with their target sites on 3′ UTRs of mRNAs[39]. Because miRNAs might result in translational inhibition without affecting the mRNA level, transcript-level approaches can miss certain targets. As a high-throughput protein platform, iTRAQ provided an easy, fast method to select certain candidate targets from the entire protein pool. Combined with expression and bioinformatic analyses, five target proteins for miR-31 and one for miR-2008 were successfully identified. Correlated miRNA and target protein expression will be investigated in our future work.

### Conclusion remarkers

Overall, the resent work firstly implicated iTRAQ into addressing possible molecular events between host-pathogen interaction in *A. japonicus*. We identified several novel pathological related proteins with down-regulated expression profiles in all examined time points, which include c-maf induced proteins, paxillin and several cytoskeleton related proteins. Also, diversified immune- and apoptosis-related proteins were also identified at each time points, revealing the temporal and spacial host-pathogen interaction progress during SUS outbreak. Importantly, six targets for our previous identified two miRNAs were also demonstrated to be regulated at translated level. The present work increase our knowledge on cellular pathways that are important for infection and pathogenesis in this non-model animals.

## Supporting Information

Table S1 Summary of the total differential expressed proteins by iTRAQ.
(XLS)

Table S2 Common and unique expressed proteins at each times.
(XLS)

Table S3 GO enrichment of the total differential expressed proteins.
(XLS)

Table S4 Putative targets for miR-31 and miR-2008 from the down-regulated expressed proteins.
(XLS)

## References

[1] Zhang C, Wang Y, Rong X, Sun H, Dong S (2004) Natural resources, culture and problems of sea cucumber worldwide. Marine Fisheries Research 25: 89–97.

[2] Wang Y, Rong X, Zhang C, Sun S (2005) Main diseases of cultured *Apostichopus japonicus*: prevention and treatment. Marine Sciences 5 29: 1–7.

[3] Wang P, Chang Y, Xu G, Song L (2005) Isolation and ultrastructure of an enveloped virus in cultured sea cucumber *Apostichopus japonicus* (Selenka). Journal Fishery Sciences of China 12(6): 766–770.

[4] Zhang C, Wang Y, Rong X (2006) Isolation and identification of causative pathogen for skin ulcerative syndrome in *Apostichopus japonicus* . Journal of Fishery China 30: 118–123.

[5] Ma Y, Xu G, Chang Y, Zhang E, Zhou W, et al (2006) Bacterial pathogens of skin ulceration disease in cultured sea cucumber *Apostichopus japonicus* (Selenka) juveniles. Journal of Dalian Ocean University Bullitin 21(1): 13–18.

[6] Bartel DP (2004) MicroRNAs: genomics, biogenesis, mechanism, and function. Cell 116: 281–297. 14744438 10.1016/s0092-8674(04)00045-5

[7] Olsen PH, Ambros V (1999) The lin-4 regulatory RNA controls developmental timing in Caenorhabditis elegans by blocking LIN-14 protein synthesis after the initiation of translation. Dev Biol 15 216(2): 671–680. 10.1006/dbio.1999.9523 10642801

[8] Seggerson K, Tang L, Moss EG (2002) Two genetic circuits repress the Caenorhabditis elegans heterochronic gene lin-28 after translation initiation. Dev Biol 2002 Mar 15 243(2): 215–225. 11884032 10.1006/dbio.2001.0563

[9] Chen C, Schaffert S, Fragoso R, Loh C (2013) Regulation of immune responses and tolerance: the microRNA perspective. Immunological Review 253(1): 112–128. 10.1111/imr.12060 PMC3684622 23550642

[10] Morita S, Horii T, Kimura M, Hatada I (2013) MiR-184 regulates insulin secretion through repression of Slc25a22. PeerJ 1: e162. 24109547 10.7717/peerj.162 PMC3792180

[11] Haasnoot J, Berkhout B (2011) RNAi and cellular miRNAs in infections by mammalian viruses. Methods in Molecular Biology 721: 23–41. 21431677 10.1007/978-1-61779-037-9_2 PMC7120436

[12] He L, Hannon GJ (2004) MicroRNAs: small RNAs with a big role in gene regulation. Nature Review Genetics 7: 522–531. 10.1038/nrg1379 15211354

[13] Li C, Feng W, Qiu L, Xia C, Su X, et al (2012) Characterization of skin ulceration syndrome associated microRNAs in sea cucumber *Apostichopus japonicus* by deep sequencing. Fish and Shellfish Immunology 33: 436–441. 22626809 10.1016/j.fsi.2012.04.013

[14] Zhang P, Li C, Zhu L, Su X, Li Y, et al (2013) De novo assembly of the sea cucumber *Apostichopus japonicus* hemocytes transcriptome to identify miRNA targets associated with skin ulceration syndrome. PLoS One 8(9): e73506. 24069201 10.1371/journal.pone.0073506 PMC3772007

[15] Rabilloud T, Chevallet M, Luche S, Lelong C (2010) Two-dimensional gel electrophoresis in proteomics: Past, present and future. Journal of Proteomics 73(11): 2064–2077. 20685252 10.1016/j.jprot.2010.05.016

[16] Thakur SS, Geiger T, Chatterjee B, Bandilla P, Fröhlich F, et al (2011) Deep and highly sensitive proteome coverage by LC-MS/MS without prefractionation. Molecular and Cell Proteomics 10(8): M110. 10.1074/mcp.M110.003699 PMC3149084 21586754

[17] Evans C, Noirel J, Ow SY, Salim M, Pereira-Medrano AG, et al (2012) An insight into iTRAQ: where do we stand now. Analytical and Bioanalytical Chemistry 404(4): 1011–1027. 22451173 10.1007/s00216-012-5918-6

[18] Herbrich SM, Cole RN, West KP Jr, Schulze K, Yager JD, et al (2013) Statistical inference from multiple iTRAQ experiments without using common reference standards. Journal of Proteome Research 12(2): 594–604. 23270375 10.1021/pr300624g PMC4223774

[19] Li Z, Lin Q, Chen J, Wu J, Lim TK, et al (2007) Shotgun identification of the structural proteome of shrimp white spot syndrome virus and iTRAQ differentiation of envelope and nucleocapsid subproteomes. Molecular and Cell Proteomics 6(9): 1609–1620. 10.1074/mcp.M600327-MCP200 17545682

[20] Lü A, Hu X, Wang Y, Shen X, Li X, et al (2014) iTRAQ analysis of gill proteins from the zebrafish (*Danio rerio*) infected with *Aeromonas hydrophila* . Fish and Shellfish Immunology 36(1): 229–239. 24269520 10.1016/j.fsi.2013.11.007

[21] Li C, Xiong Q, Zhang J, Ge F, Bi L (2012) Quantitative proteomic strategies for the identification of microRNA targets. Expert Review of Proteomics 9(5): 549–559. 23194271 10.1586/epr.12.49

[22] Ou M, Zhang X, Dai Y, Gao J, Zhu M, et al.. (2014) Identification of potential microRNA-target pairs associated with osteopetrosis by deep sequencing, iTRAQ proteomics and bioinformatics. European Journal of Human Genetics doi: 10.1038/ejhg.2013.221. 10.1038/ejhg.2013.221 PMC3992578 24084574

[23] Zhou Z, Dong Y, Sun H, Yang AF, Chen Z, et al (2014) Transcriptome sequencing of sea cucumber (*Apostichopus japonicus*) and the identification of gene-associated markers. Molecular Ecology Resources14(1): 127–138. 10.1111/1755-0998.12147 23855518

[24] Shao Y, Li C, Ou C, Zhang P, Lu Y, et al (2013) Divergent metabolic responses of *Apostichopus japonicus* suffered from skin ulceration syndrome and pathogen challenge. Journal of Agricultural and Food Chemistry 61(45): 10766–10771. 24127639 10.1021/jf4038776

[25] Bradford MM (1976) A rapid and sensitive method for the quantitation of microgram quantities of protein utilizing the principle of protein-dye binding. Analytical Biochemistry 72: 248–254. 942051 10.1016/0003-2697(76)90527-3

[26] Shilov IV, Seymour SL, Patel AA, Loboda A, Tang WH, et al (2007) The Paragon Algorithm, a next generation search engine that uses sequence temperature values and feature probabilities to identify peptides from tandem mass spectra. Molecular and Cellular Proteomics 6(9): 1638–1655. 17533153 10.1074/mcp.T600050-MCP200

[27] Chen M, Zhang X, Liu J, Storey KB (2013) High-throughput sequencing reveals differential expression of miRNAs in intestine from sea cucumber duringaestivation. PLoS One 15 8(10): e76120. 10.1371/journal.pone.0076120 PMC3797095 24143179

[28] Zhu L, Li C, Su X, Guo C, Wang Z, et al (2013) Identification and assessment of differentially expressed genes involved in growth regulation in Apostichopus japonicus. Genet Mol Res 20 12(3): 3028–3037. 10.4238/2013.August.20.4 24065658

[29] Di Pietro G, Magno LA, Rios-Santos F (2010) Glutathione S-transferases: an overview in cancer research. Expert Opinion on Drug Metabolism and Toxicology 6(2): 153–170. 20078251 10.1517/17425250903427980

[30] Saranya Revathy K, Umasuthan N, Choi CY, Whang I, Lee J (2012) First molluscan theta-class Glutathione S-Transferase: identification, cloning, characterization and transcriptional analysis post immune challenges. Comparative Biochemistry and Physiology Part B: Biochemistry and Molecular Biology 162(1–3): 10–23. 10.1016/j.cbpb.2012.02.004 22390916

[31] Ren Y, Ding Q, Zhang X (2014) Ficolins and infectious diseases. Virological Sinica 29(1): 25–32. 10.1007/s12250-014-3421-2 PMC8206374 24452543

[32] Lopez J, Hesling C, Prudent J, Popgeorgiev N, Gadet R, et al (2012) Src tyrosine kinase inhibits apoptosis through the Erk1/2- dependent degradation of the death accelerator Bik. Cell Death and Differentiation 19(9): 1459–1469. 22388352 10.1038/cdd.2012.21 PMC3422470

[33] Lietzén N, Ohman T, Rintahaka J, Julkunen I, Aittokallio T, et al (2011) Quantitative subcellular proteome and secretome profiling of influenza A virus-infected human primary macrophages. PLoS Pathogens 7(5): e1001340. 21589892 10.1371/journal.ppat.1001340 PMC3093355

[34] Tsuruta Y, Park YJ, Siegal GP, Liu G, Abraham E (2007) Involvement of vitronectin in lipopolysaccaride-induced acute lung injury. Journal of Immunology 179(10): 7079–7086. 10.4049/jimmunol.179.10.7079 17982099

[35] Bae HB, Zmijewski JW, Deshane JS, Zhi D, Thompson LC, et al (2012) Vitronectin inhibits neutrophil apoptosis through activation of integrin-associated signaling pathways. American Journal of Respiratory Cell and Molecular Biology 46(6): 790–796. 22281987 10.1165/rcmb.2011-0187OC PMC3380283

[36] Subauste MC, Pertz O, Adamson ED, Turner CE, Junger S (2004) Vinculin modulation of paxillin-FAK interactions regulates ERK to control survival and motility. The Journal of Cell Biology 165(3): 371–381. 15138291 10.1083/jcb.200308011 PMC2172187

[37] Magro AM, Magro AD, Cunningham C, Miller MR (2007) Down-regulation of vinculin upon MK886-induced apoptosis in LN18 glioblastoma cells. Neoplasma 54(6): 517–526. 17949236 PMC4320946

[38] Peng X, Nelson ES, Maiers JL, DeMali KA (2011) New insights into vinculin function and regulation. Innternational Review of Cell and Molecular Biology 287: 191–231. 10.1016/B978-0-12-386043-9.00005-0 PMC4426885 21414589

[39] Uhlmann S, Mannsperger H, Zhang J, Horvat EÁ, Schmidt C, et al (2012) Global microRNA level regulation of EGFR-driven cell-cycle protein network in breast cancer. Molecular Systems Biology 8: 570. 22333974 10.1038/msb.2011.100 PMC3293631

